# Human herpesvirus 6 infection impairs Toll-like receptor signaling

**DOI:** 10.1186/1743-422X-7-91

**Published:** 2010-05-10

**Authors:** Yuichi Murakami, Kazushi Tanimoto, Hiroshi Fujiwara, Jun An, Koichiro Suemori, Toshiki Ochi, Hitoshi Hasegawa, Masaki Yasukawa

**Affiliations:** 1 Departmemt of Bioregulatory Medicine, Ehime University Graduate School of Medicine, Toon, Ehime 791-0295, Japan; 2 Proteo-Medicine Research Center, Ehime University, Toon, Ehime 791-0295, Japan

## Abstract

Human herpesvirus 6 (HHV-6) has a tropism for immunocompetent cells, including T lymphocytes, monocytes/macrophages, and dendritic cells (DCs) suggesting that HHV-6 infection affects the immunosurveillance system. Toll-like receptor (TLR) system plays an important role in innate immunity against various pathogens. In the present study, we investigated the effect of HHV-6 infection on the expression and intracellular signaling of TLRs in DCs. Although expression levels of TLRs were not decreased or slightly elevated following HHV-6 infection, the amounts of cytokines produced following stimulation with ligands for TLRs appeared to be dramatically decreased in HHV-6-infected DCs as compared to mock-infected DCs. Similarly, phosphorylation levels of TAK-1, IκB kinase, and IκB-α following stimulation of HHV-6-infected DCs with lipopolysaccharide, which is the ligand for TLR4, appeared to be decreased. These data show that HHV-6 impairs intracellular signaling through TLRs indicating the novel mechanism of HHV-6-mediated immunomodulation.

## Findings

Human herpesvirus 6 (HHV-6) is known as a causative agent of exanthem subitum, and reactivation of HHV-6 in adults causes various clinical manifestations [1,2]. HHV-6 can preferentially infect immunocompetent cells and induces various immunobiological alterations [3-12]. Therefore, HHV-6 is recognized as one of the important viruses that modulate immune responses.

Toll-like receptors (TLRs) are key molecules of the innate immune system [13]. A subset of TLRs recognizes components of microorganisms and induces innate immune responses. After recognition of ligands, TLRs activate their intrinsic signaling pathways, resulting in activation of the transcription factor nuclear factor-κB (NF-κB), which controls the expression of inflammatory cytokine genes [14,15]. HHV-6 alters the regulation of innate immunity as well as adaptive immunity. In the light of these facts, it seems important to clarify the effects of HHV-6 infection on the TLR system. We therefore investigated the effects of HHV-6 infection on the expression and functions of TLRs in DCs.

The Z29 strain of HHV-6B was mainly used in the present study, because HHV-6B is more prevalent than HHV-6A in the general population. Immature DCs were generated from peripheral blood monocytes by culturing them in the presence of GM-CSF and IL-4, as described previously [8]. Immature DCs were inoculated with HHV-6 at an approximate multiplicity of infection of 1 50% tissue culture infective dose. HHV-6-inoculated DCs were cultured for 3 days and used for experiments. More than 95% of HHV-6-infected and mock-infected DCs were viable when used for experiments.

Expression of mRNA for TLRs1-10 in HHV-6-infected and mock-infected DCs was examined by semi-quantitative reverse transcription-polymerase chain reaction (RT-PCR) [16]. Sequences of the primers for PCR are shown in the additional file 1.

Cytokine production by DCs was examined as follows. After 3 days of HHV6 inoculation, DCs were cultured for 24 hours in RPMI 1640 medium supplemented with 10% fetal calf serum and poly(I:C) (a ligand for TLR 3; Invitrogen, San Diego, CA, USA) at 25 μg/ml, lipopolysaccharide (LPS) (a ligand for TLR 4; Sigma, St Louis, MO, USA) at 100 ng/ml, or imidazoquinoline (a ligand for TLR7; Invitrogen) at 5 mg/ml. The culture supernatants were then harvested, and the amounts of cytokines they contained were measured by flow cytometry using a Cytometric Bead Array System (BD Biosciences, San Diego, CA, USA) and enzyme-linked immunosorbent assay (Biosource Europe S.A., Nivelles, Belgium).

The binding of LPS to HHV-6-infected and mock-infected DCs was examined quantitatively by flow cytometry using fluorescent LPS conjugate (Alexa Fluor^® ^488) (Molecular Probes, Eugene, OR, USA).

Western blotting was performed by a standard method using the following antibodies; anti-TLR4 (BioChain, Hayward, CA, USA), anti-MyD88 (ProSci, Poway, CA, USA), anti-TRAF6 (Santa Cruz Biotechnology, Santa Cruz, CA, USA), anti-TAK-1 (Cell Signaling Technology, Danvers, MA, USA), anti-phosphorylated IκB kinase α/β (IKKα/β) (Cell Signaling Technology), anti-phosphorylated IκB-α (Cell Signaling Technology), and anti-β-actin (Sigma).

We first confirmed HHV-6 infection in DCs. We and other investigators previously reported that HHV-6 can infect human DCs and modulates the expression of various surface molecules including CD80, CD83, CD86, and DC-SIGN [8,9,17]. As shown in the additional file 2, expression of HHV-6 immediate early and late genes was detected in HHV-6-inoculated DCs. In addition, two-color flow cytometry showed that HHV-6 antigen expression was present in more than half of the DCs inoculated with HHV-6. HHV-6 antigen expression was detected in DCs in which CD80 expression was up-regulated, as we have reported previously [8] (Additional file 3). These data confirmed that HHV-6 was able to infect DCs under our experimental conditions.

**Figure 1:**
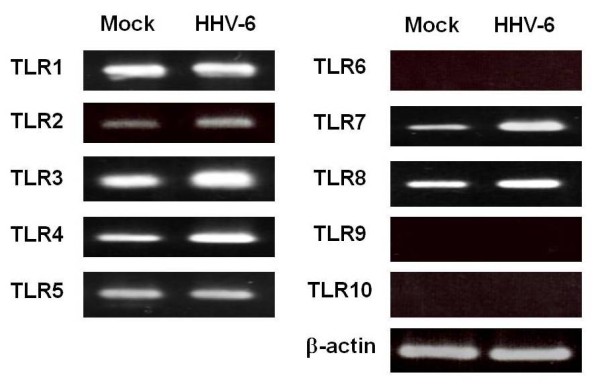
**RT-PCR analysis of TLR mRNAs in mock-infected and HHV-6-infected DCs**. Semi-quantitative RT-PCR reveals that expression levels of mRNAs for TLR3, TLR4, and TLR7 are slightly higher in HHV-6-infected DCs than in mock-infected DCs.

We screened the TLR1-10 expression in HHV-6-infected DCs and compared it with that in mock-infected DCs. As shown in Figure 1, semi-quantitative RT-PCR revealed that expression of mRNAs for TLR3, TLR4, and TLR7 appeared to be slightly increased in HHV-6-infected DCs as compared with mock-infected DCs.

**Figure 2:**
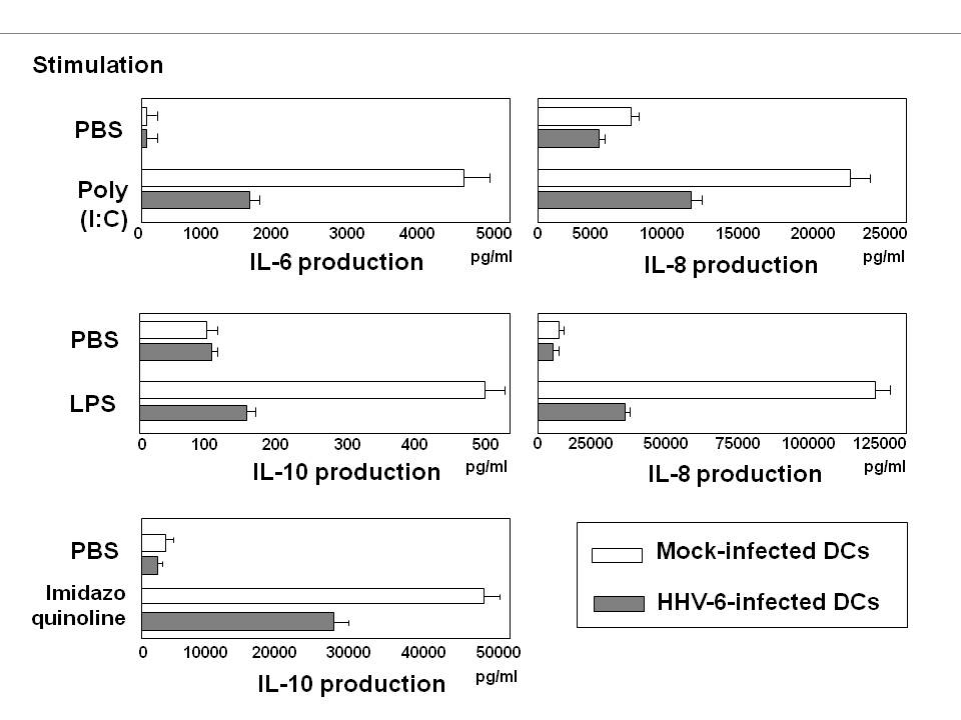
**Downregulation of cytokine production by stimulation with TLR ligand in DCs after infection with HHV-6**. The production of cytokines by HHV-6-infetced DCs and mock-infected DCs was examined as detailed in the text. The amounts of cytokines produced by HHV-6-infected DCs after stimulation with the TLR3 ligand poly(I:C), the TLR4 ligand LPS, and the TLR7 ligand imidazoquinoline are all lower than those produced by mock-infected DCs that were stimulated with TLR ligands.

We next examined cytokine production by HHV-6-infected and mock-infected DCs in response to stimulation with TLR ligands. TLR3, TLR4, and TLR7 were selected for this experiment, because expression of these TLRs seemed to be increased after infection with HHV-6, as shown in Figure 1. As shown in Figure 2, the amounts of IL-6 and IL-8 produced by DCs stimulated with poly(I:C), a TLR3 ligand, after infection with HHV-6 appeared to be significantly lower than those produced by mock-infected DCs. Similarly, the production of IL-10 and IL-8 by HHV-6-infected DCs in response to stimulation with LPS, a TLR4 ligand, was markedly impaired in comparison with mock-infected DCs. The amount of IL-8 produced by HHV-6-infected DCs stimulated with a TLR7 ligand, imidazoquinoline, was also decreased as compared with that produced by TLR7 ligand-stimulated mock-infected DCs. The same experiments were performed three times and similar data were obtained.

**Figure 3:**
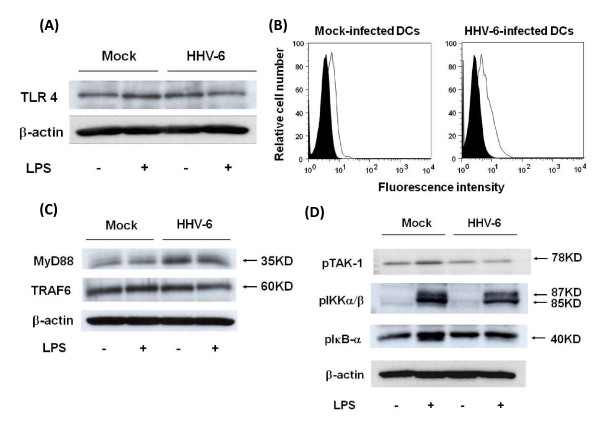
**Impairment of TLR4 signaling in HHV-6-infected DCs**. (A) Western blotting reveals that the expression level of TLR4 protein in HHV-6-infected DCs, which were not stimulated with LPS, is slightly higher than that in mock-infected DCs. (B) Flow cytometric analysis using fluorescent LPS conjugate reveals that the amount of LPS bound to HHV-6-infected DCs is slightly higher than that on mock-infected DCs, suggesting that the expression level of TLR4 molecules on DCs is increased after infection with HHV-6. (C) Western blotting reveals that the expression levels of MyD88 and TRAF6 proteins in HHV-6-infected DCs, which are not stimulated with LPS, are slightly higher than those in mock-infected DCs. (D) Western blotting reveals that phosphorylation levels of TAK-1, IKKα/β and IκB-α in HHV-6-infected DCs after stimulation with LPS are significantly lower than those in LPS-stimulated mock-infected DCs.

We further examined the mechanisms of impaired cytokine production by HHV-6-infected DCs, focusing on TLR4. First, expression of the TLR4 molecule on HHV-6-infected and mock-infected DCs was examined by Western blotting of cell lysates and flow cytometry to detect the binding of fluorescent LPS conjugate. As shown in Figures 3A and 3B, the level of TLR4 expression on HHV-6-infected DCs unstimulated with LPS appeared to be slightly higher than that on mock-infected DCs.

The intracellular signaling system of TLR4 in HHV-6-infected and mock-infected DCs was further examined. First, it appeared that the amount of MyD88, an adaptor molecule required for signal transduction through TLRs, was slightly higher in HHV-6-infected DCs than in mock-infected DCs, parallel to the TLR4 expression level. Similarly, the expression level of TRAF6, another TLR adaptor molecule, did not differ significantly, or was slightly increased, in HHV-6-infected as compared with mock-infected DCs (Figure 3C). In contrast, phosphorylation levels of TAK-1, IKKα/β, and IκB-α, which are important molecules for NF-κB activation [18], in HHV-6-infected DCs after stimulation with LPS appeared to be significantly lower than those in LPS-stimulated mock-infected DCs (Figure 3D). The same experiments were performed twice and similar data were obtained. These data reveal that HHV-6 impairs signal transduction of TLRs.

In the present study, we demonstrated that although the expression of TLRs and their adaptor molecules was only slightly increased, cytokine production by DCs in response to stimulation with TLR ligands was severely impaired after infection with HHV-6. In contrast, phosphorylation levels of TAK-1, IKKα/β and IκB-α appeared to be significantly decreased in HHV-6-infected DCs as compared with mock-infected DCs. NF-κB activation resulting in production of inflammatory cytokines depends on phosphrylation of IκB, which is induced by activation of the IKK complex [18,19]. Activation of IKKs depends on their phosphorylation, which results in conformational change and kinase activity [20-22]. It is also noteworthy that impaired cytokine production is not restricted in the TLR4 system but is also detected in the signal pathways of other TLRs. Therefore, these findings suggest that impairment of TLR4 signaling in HHV-6-infected DCs is due to blocking not upstream, but downstream in the signal pathway.

Recently, various effects of viral infection on expression and signaling of TLRs have been reported. Chen *et al. *have recently reported that vaccinia virus virulence factor B14 can directly bind to the IKK complex and inhibit phosphorylation of IKKβ[23]. This results in impairment of IκB-α degradation and inhibition of NF-κB activation. In the present study, it was found that inoculation with inactivated HHV-6 did not induce impairment of TLR signaling, i.e., the amounts of cytokines produced by HHV-6-infected and inactivated HHV-6-inoculated DCs following stimulation with TLR ligands were not significantly different (data not shown). Therefore, HHV-6 gene product(s) produced *de novo *in HHV-6-infected DCs might associate with the TAK-1 or IKK complex directly or indirectly, resulting in inhibition of IKK activation, as is the case for vaccinia virus infection. It has also been reported that M45 protein of murine cytomegalovirus, which, like HHV-6, is a β-herpesvirus, inhibits the RIP1-mediated activation of NF-κB in response to TLR3 stimulation [24]. This finding suggests that impairment of TLR signaling might be the common strategy of immune evasion by β-herpesviruses.

Viruses alter cell functions via mainly direct infection; however, indirect mechanisms are also responsible for virus-mediated immune-modulation. We previously reported that HHV-6 infection mediates apoptosis in HHV-6-uninfected T cells through a bystander effect [25]. In the present study, it was not clarified whether a direct or a bystander effect plays an important role in impairment of the innate immune response in HHV-6 infection. Further study will be needed to clarify this issue.

In summary, we have demonstrated for the first time that the intracellular signaling pathway through TLRs is severely impaired by HHV-6 infection. Since the TLR system is essential for recognition of various pathogens and generation of innate immunity, disruption of TLR-mediated signaling seems to be an effective strategy by which viruses can evade the immunosurveillance system.

## Competing interests

The authors declare that they have no competing interests.

## Authors' contributions

YM, KT, JA, KS, and TO carried out the experiments. HF and HH participated in the design of the study and supported performing experiments. MY designed the research, wrote and edited the paper, and provided financial support. All authors read and approved the final manuscript.

## Supplementary Material

Additional file 1 **Sequences of the primers for RT-PCR**. Expression of mRNA for TLRs1-10 and β-actin in HHV-6-infected and mock-infected DCs was examined by RT-PCR using the primers shown here. Click here for file

Additional file 2 **Expression of HHV-6 mRNA in DCs**. cDNAs synthesized from HHV-6-infected cord blood cells (lane 1), distilled water only (lane 2), mock-infected DCs (lane 3), and HHV-6-infected DCs on day 5 after inoculation (lane 4) were amplified using primers corresponding to the HHV-6 immediate-early and late genes and primers corresponding to the β-actin gene. Click here for file

Additional file 3 **Flow cytometric analysis of HHV-6 antigen expression in DCs**. Mock-infected DCs and HHV-6-infected DCs on day 5 after inoculation were stained with anti-HHV-6 gB monoclonal antibody and anti-CD80 monoclonal antibody. Click here for file
