# Supplementary figures and images for: Human herpesvirus 6 infection impairs Toll-like receptor signaling
Source: Virol J. 2010 May 10;7:91. doi: 10.1186/1743-422X-7-91 (PMC2874541; doi:10.1186/1743-422X-7-91)

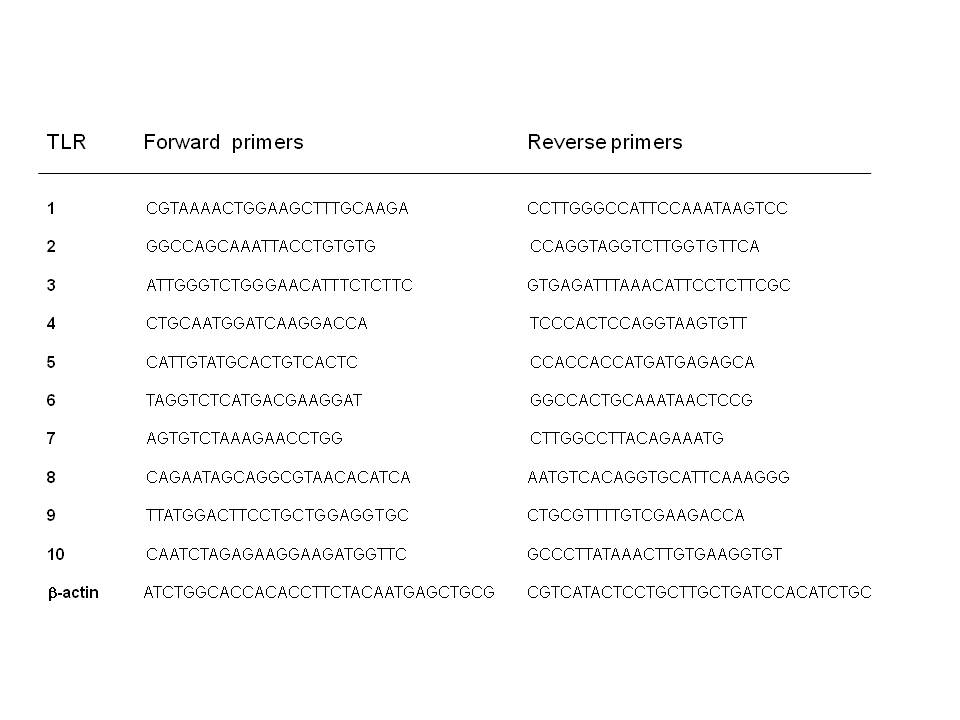

Supplement: Additional file 1 — Sequences of the primers for RT-PCR. Expression of mRNA for TLRs1-10 and β-actin in HHV-6-infected and mock-infected DCs was examined by RT-PCR using the primers shown here. [file 1743-422X-7-91-S1.JPEG]

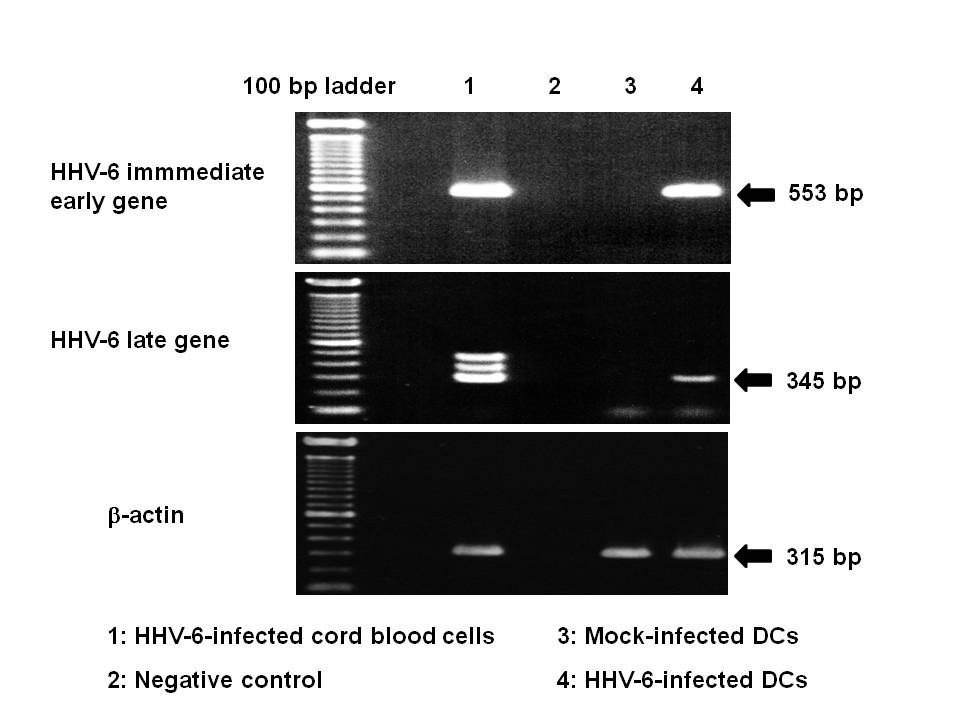

Supplement: Additional file 2 — Expression of HHV-6 mRNA in DCs. cDNAs synthesized from HHV-6-infected cord blood cells (lane 1), distilled water only (lane 2), mock-infected DCs (lane 3), and HHV-6-infected DCs on day 5 after inoculation (lane 4) were amplified using primers corresponding to the HHV-6 immediate-early and late genes and primers corresponding to the β-actin gene. [file 1743-422X-7-91-S2.JPEG]

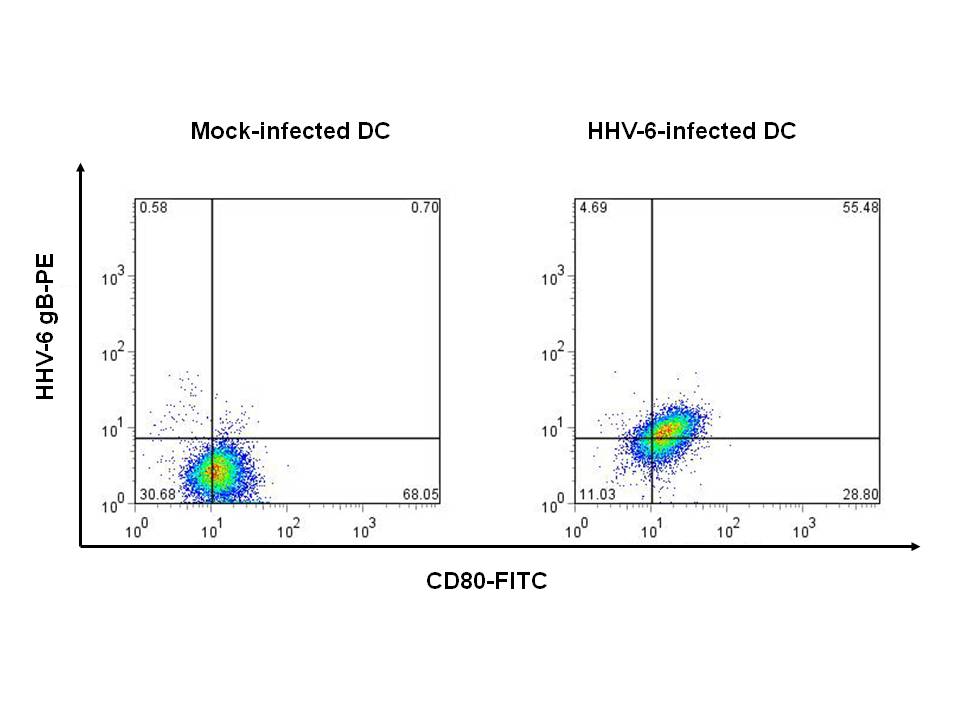

Supplement: Additional file 3 — Flow cytometric analysis of HHV-6 antigen expression in DCs. Mock-infected DCs and HHV-6-infected DCs on day 5 after inoculation were stained with anti-HHV-6 gB monoclonal antibody and anti-CD80 monoclonal antibody. [file 1743-422X-7-91-S3.JPEG]
